# BPR0C261, An Analogous of Microtubule Disrupting Agent D-24851 Enhances the Radiosensitivity of Human Non-Small Cell Lung Cancer Cells via p53-Dependent and p53-Independent Pathways

**DOI:** 10.3390/ijms232214083

**Published:** 2022-11-15

**Authors:** Jyh-Der Leu, Shih-Ting Lin, Chiung-Tong Chen, C.-Allen Chang, Yi-Jang Lee

**Affiliations:** 1 Division of Radiation Oncology, Taipei City Hospital RenAi Branch, Taipei 106, Taiwan; 2 Institute of Neuroscience, National Chengchi University, Taipei 116, Taiwan; 3 Department of Biomedical Imaging and Radiological Sciences, National Yang Ming Chiao Tung University, Taipei 11221, Taiwan; 4 Institute of Biotechnology and Pharmaceutical Research, National Health Research Institutes, Zhunan 350, Taiwan; 5 Cancer Progression Research Center, National Yang Ming Chiao Tung University, Taipei 11221, Taiwan

**Keywords:** NSCLC, BPR0C261, radiosensitivity, DNA damage, microtubule inhibitor, p53, PTEN

## Abstract

(1) Destabilization of microtubule dynamics is a primary strategy to inhibit fast growing tumor cells. The low cytotoxic derivative of microtubule inhibitor D-24851, named BPR0C261 exhibits antitumor activity via oral administration. In this study, we investigated if BPR0C261 could modulate the radiation response of human non-small cell lung cancer (NSCLC) cells with or without p53 expression. (2) Different doses of BPR0C261 was used to treat human NSCLC A549 (p53+/+) cells and H1299 (p53−/−) cells. The cytotoxicity, radiosensitivity, cell cycle distribution, DNA damage, and protein expression were evaluated using an MTT assay, a colony formation assay, flow cytometry, a comet assay, and an immunoblotting analysis, respectively. (3) BPR0C261 showed a dose-dependent cytotoxicity on A549 cells and H1299 cells with IC_50_ at 0.38 μM and 0.86 μM, respectively. BPR0C261 also induced maximum G_2_/M phase arrest and apoptosis in both cell lines after 24 h of treatment with a dose-dependent manner. The colony formation analysis demonstrated that a combination of low concentration of BPR0C261 and X-rays caused a synergistic radiosensitizing effect on NSCLC cells. Additionally, we found that a low concentration of BPR0C261 was sufficient to induce DNA damage in these cells, and it increased the level of DNA damage induced by a fractionation radiation dose (2 Gy) of conventional radiotherapy. Furthermore, the p53 protein level of A549 cell line was upregulated by BPR0C261. On the other hand, the expression of PTEN tumor suppressor was found to be upregulated in H1299 cells but not in A549 cells under the same treatment. Although radiation could not induce PTEN in H1299 cells, a combination of low concentration of BPR0C261 and radiation could reverse this situation. (4) BPR0C261 exhibits specific anticancer effects on NSCLC cells by the enhancement of DNA damage and radiosensitivity with p53-dependent and p53-independent/PTEN-dependent manners. The combination of radiation and BPR0C261 may provide an important strategy for the improvement of radiotherapeutic treatment.

## 1. Introduction

Human non-small cell lung cancer (NSCLC) is the primary type of lung cancer globally. In the United States, 236,740 new cases and 350 deaths per day are reported for lung cancer, which is still the leading cause of cancer death in 2022 [1]. The good news is that the incidence and the mortality of NSCLC are decreasing because of the increasing knowledge on potential risk factors [2]. According to the stage of NSCLC, the treatments range from pure surgery, chemo-radiotherapy, immunotherapy, and targeted drug therapy [3]. The adjuvant chemo-radiotherapy is the most traditional and common method for NSCLC therapy in different stages [4,5]. However, effective therapeutic approaches for NSCLC remain to be developed.

Hundreds of *N*-heterocyclic indolyl glyoxylamides have been synthesized and evaluated for their anticancer activity, including cancer cells of murine leukemia, human gastric, breast, and uterus sources [6]. These compounds are structurally analogous to N-(pyridin-4-yl)-[1-(4-chlorbenzyl)-indol-3-yl]-glyoxyl-amid (D-24851) that functions as a microtubule inhibitor containing oral antitumor activity in vivo, as well as multidrug-resistance tumor cells [7]. D-24851 has been reported to induce apoptosis in malignant glioma cells without active p53, a well-known tumor suppressor protein [8]. Interestingly, two compounds (BPR0C123 and BPR0C259) of *N*-heterocyclic indolyl glyoxylamides exhibited different cytotoxicity on human NSCLC cell lines, but also induced p53-independent apoptosis and radiosensitivity to different levels [9]. BPR0C261 is identified as the most cytotoxic molecules against a broad spectrum of mammalian cancer cells in this series of compounds [6]. The antitumor actions of BPR0C261 include antimitosis and anti-angiogenesis in vivo [10]. Because BPR0C261 can also be administrated orally and extend the lifespan of tumor-bearing mice, it is interesting to be considered in clinical applications [10]. Whether BPR0C261 also induces cytotoxicity and radiosensitivity in NSCLC with or without p53 tumor suppressor gene is still unclear.

The homozygous mutation of the p53 tumor suppressor gene is engaged in 50–60% of human cancers [11]. The biochemical role of the p53 gene is to encode a transcription factor and transactivate downstream genes for apoptosis, DNA repair, senescence, and cell cycle arrest [12]. A recent report focused on the crosstalk between p53 and immunity and demonstrated that mutant p53 could suppress innate immunity to promote tumorigenesis [13]. In addition, p53 can transcriptionally suppress the expression of vascular endothelial growth factor (VEGF) and induce the production of arrestin to inhibit angiogenesis in human tumor [14,15]. Although p53 is known to be important for the induction of apoptosis in cancer cells exposed to genotoxicity, the p53-independent pathways are also frequently reported to mediate drug-induced apoptosis [16,17,18]. For instance, the PTEN (phosphatase and tensin homolog) tumor suppressor has been reported to be involved in p53-independnet apoptosis [19]. PTEN is a negative regulator of the phosphatidylinositol -3 kinase (PI3K)/Akt/mTOR pathway that phosphorylates phosphatidylinositol (4,5)-trisphosphate (PIP_2_) to PIP_3_ to trigger a series of signal transduction and promote cell survival, cell cycle progression, cell growth, and angiogenesis [20,21]. Interestingly, the promoter of the PTEN gene harbors a p53-binding element that can be directly regulated by wild-type p53, although PTEN is constitutively expressed through a p53-independnet element [22]. PTEN mutations are mainly discovered in endometrial carcinomas and glioblastomas and are associated with 13.5% of human cancers [23,24]. PTEN can inhibit the cell cycle progression and promote apoptosis in NSCLC cells [25]. Moreover, a combination of radiation and paclitaxel has been reported to trigger a PTEN–PI3K–Akt–Bax signaling cascade in NSCLC xenograft tumors and suppress tumor growth in the absence of functional p53 [26]. However, the effect of BPR0C261 on the expression of p53 and/or PTEN tumor suppressors in NSCLC cells remains to be investigated.

In this study, we combine BPR0C261 and radiation for the treatment of NSCLC cells and compare the results with the individual effect of both methods. The cell cycle, DNA damage, radiosensitivity, and cell apoptosis are measured and evaluated. We also demonstrate that p53 expression is significant in NSCLC cells containing wild-type p53, while PTEN becomes dominant in those with deleted p53. The association of tumor suppressors with the BPR0C261-enhanced radiosensitivity is discussed.

## 2. Results

### 2.1. Effects of BPR0C261 on NSCLC Cells

**Figure 1:**
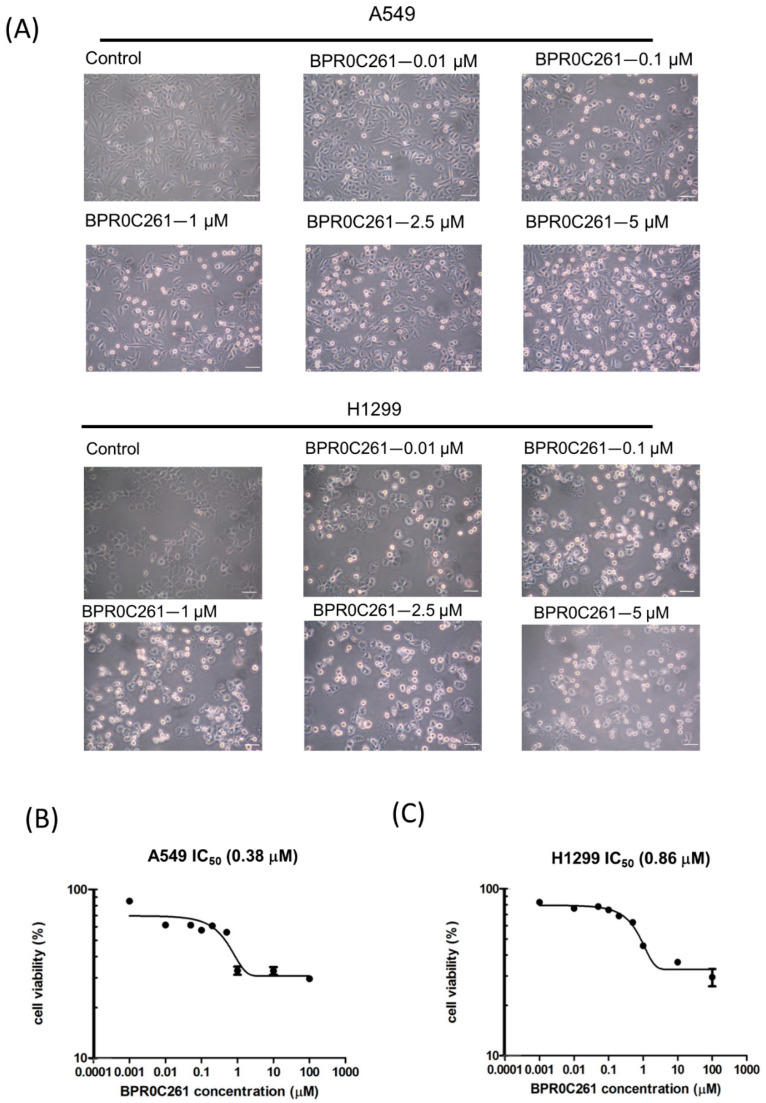
Cytotoxic effects of BPR0C261 on NSCLC cells. (**A**) Microscopic visualization of cell morphological changes by BPR0C261. Magnification: 40×; scale bar: 100 m. (**B**,**C**) The MTT assay for estimation of IC_50_ of BPR0C261 on A549 cells and H1299 cells after 24 h of treatment, respectively.

First, NSCLC cells were treated with increased concentrations of BPR0C261 and the drug effects on cells were detected by morphological change and the MTT assay. Starting from 0.01 μM BPR0C261, an increase of round-up cells could be detected in A549 cells and H1299 cells exposed to increased concentrations of the drug for 12 h (Figure 1A). We also measured the IC_50_ of BPR0C261 by a dose-dependent experiment and showed that A549 cells were more sensitive to the drug than H1299 cells (Figure 1B). The IC_50_ of BPR0C261 on A549 cells and H1299 cells were 0.38 μM and 0.86 μM, respectively.

### 2.2. Effects of BPR0C261 on the Redistribution of Cell Cycle

**Figure 2:**
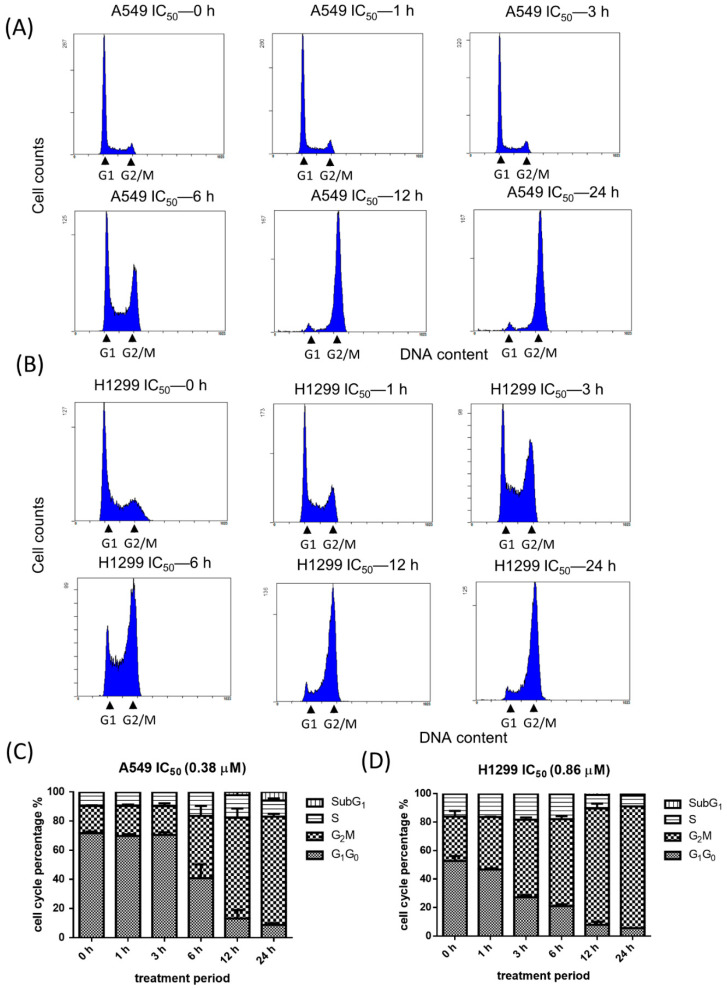
Analysis of cell cycle distribution by flow cytometry after NSCLC cells were treated with BPR0C261. (**A**) A549 cells and (**B**) H1299 cells were treated with their corresponding IC_50_ of BPR0C261 with a time-dependent cell cycle redistribution. (**C**,**D**) Quantification of percentage of each cell cycle phase in A549 cells and H1299 cells, respectively. Each datum represents the mean of three independent experiments ± S.D.

We next examined the cell cycle of A549 cells and H1299 cells treated with BPR0C261. Through the flow cytometry, the percentage of cells at each phase after BPR0C261 treatment was indicated from 6 h to 24 h. The results showed that BPR0C261 caused significant G_2_/M phase arrest in both A549 cells and H1299 using individual IC_50_ (Figure 2A,B). Notably, A549 cells harbored wild-type p53 while H1299 cells contained null-p53, and this was associated with different patterns of cell cycle progression treated with BPR0C261 for 3 h to 6 h. That is, the G_2_/M phase arrest of H1299 cells happened three hours earlier than that of A549 cells treated with BPR0C261. Nevertheless, the highest G2/M phase arrest was still detected in both cell lines after 12 h of treatment. The percentage of each phase of the cell cycle was also quantified in both cell lines treated with the corresponding IC_50_ of BPR0C261 (Figure 2C,D).

### 2.3. Effects of High Concentration of BPR0C261 on Induction of Sub-G1 Population in NSCLC Cells 

**Figure 3:**
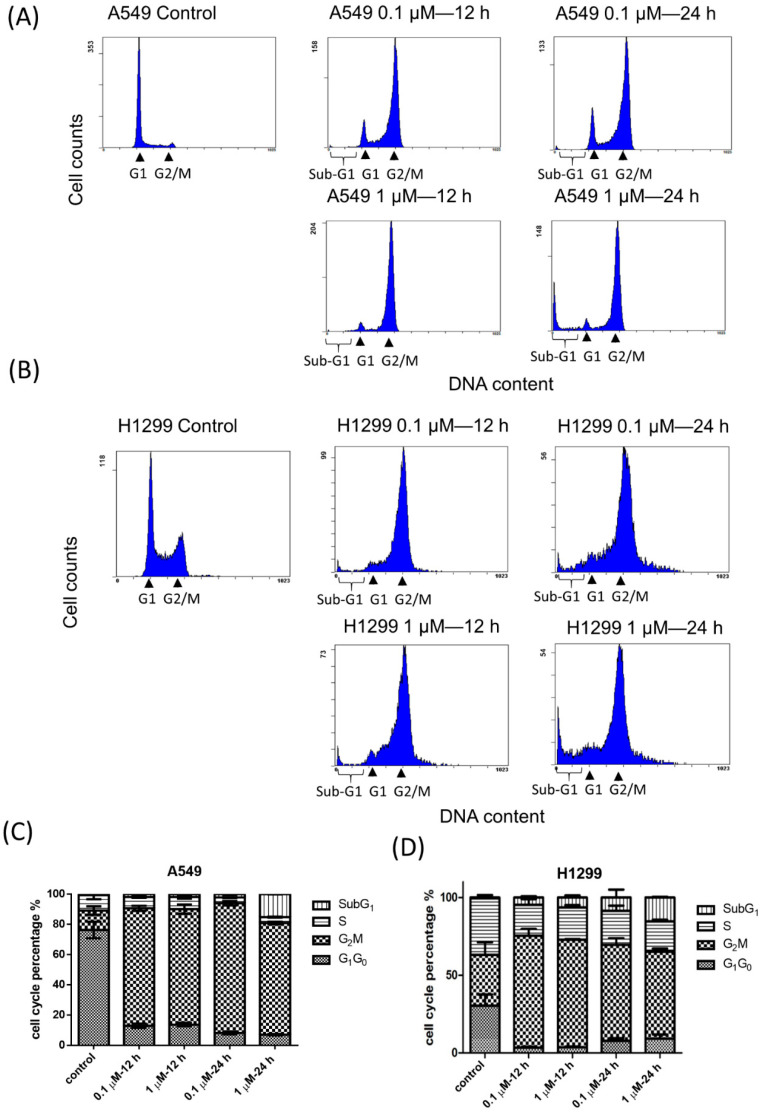
Comparison of sub-G1 phase of NSCLC cells treated with high and low concentration of BPR0C261. The sub-G1 phase of (**A**) A549 cells and (**B**) H1299 cells treated with 0.1 M and 1 M of BPR0C261 for 12 h and 24 h. (**C**,**D**) Quantification of percentage of sub-G1 phase in A549 cells and H1299 cells treated with BPR0C261, respectively. Each datum represents the mean of three independent experiments ± S.D.

We next examined cell cycle redistribution including the percentage of sub-G1 phase of NSCLC cells treated with BPR0C261 at 0.1 M and 1 M that were below and over IC_50_, respectively. Compared to 1 M BPR0C261, 0.1 M BPR0C261 did not induce significant percentage of sub-G1 phase in A549 cells after 24 h of treatment, although both concentrations still induced a significant G2/M phase arrest after 12 h of treatment (Figure 3A). A similar phenomenon was also detected in H1299 cells treated with BPR0C261, but a low concentration of BPR0C261 appeared to induce significant sub-G1 phase compared to untreated control (Figure 3B). The results of DNA histograms of A549 cells and H1299 cells treated with different concentrations of BPR0C261 were quantified (Figure 3C,D). 

### 2.4. The Combination Treatment of Radiation and BPR0C261 Decreased the Survival Fraction in NSCLC Cells

**Figure 4:**
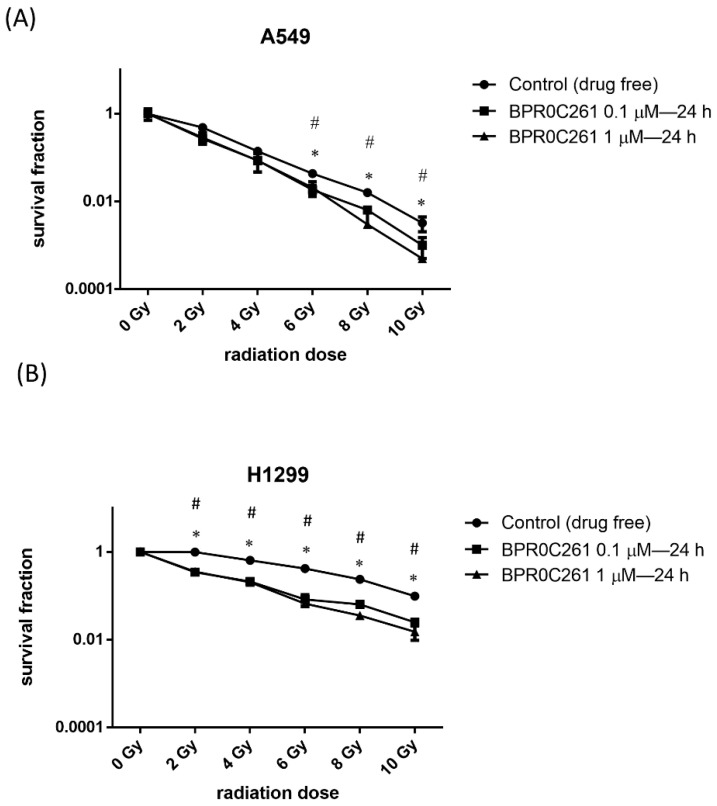
Effect of BPR0C261 on radiosensitivity of NSCLC cells. The survival fraction of (**A**) A549 cells and (**B**) H1299 cells exposed to different doses of X-rays after treatment with BPR0C261. *: *p* < 0.05 for 0.1 μM BPR0C261 treatment compared to radiation alone. #: *p* < 0.05 for 1 μM BPR0C261 treatment compared to radiation alone.

The surviving fraction of A549 and H1299 were evaluated after a treatment with BPR0C261 or radiation alone and then the combination of the two treatments using the colony formation assay. The concentrations of BPR0C261 used were 0.1 μM and 1 μM, which are lower or higher than the IC_50_, respectively. Compared to the radiation treatment alone, BPR0C261 enhanced the radiosensitivity of both A549 cells and H1299 cells (Figure 4 and Supplementary Material S1). Intuitively, the higher the concentration of BPR0C261, the lower the cell survival rate. Using the method of Valeriote and Carpentier (see Materials and Methods), the combination of BPR0C261 and radiation showed a synergistic effect on NSCLC cells compared to the treatment with radiation alone (Table 1 and Table 2). 

### 2.5. Effects of BPR0C261 and Radiation on Induction of DNA Damage in NSCLC Cells

**Figure 5:**
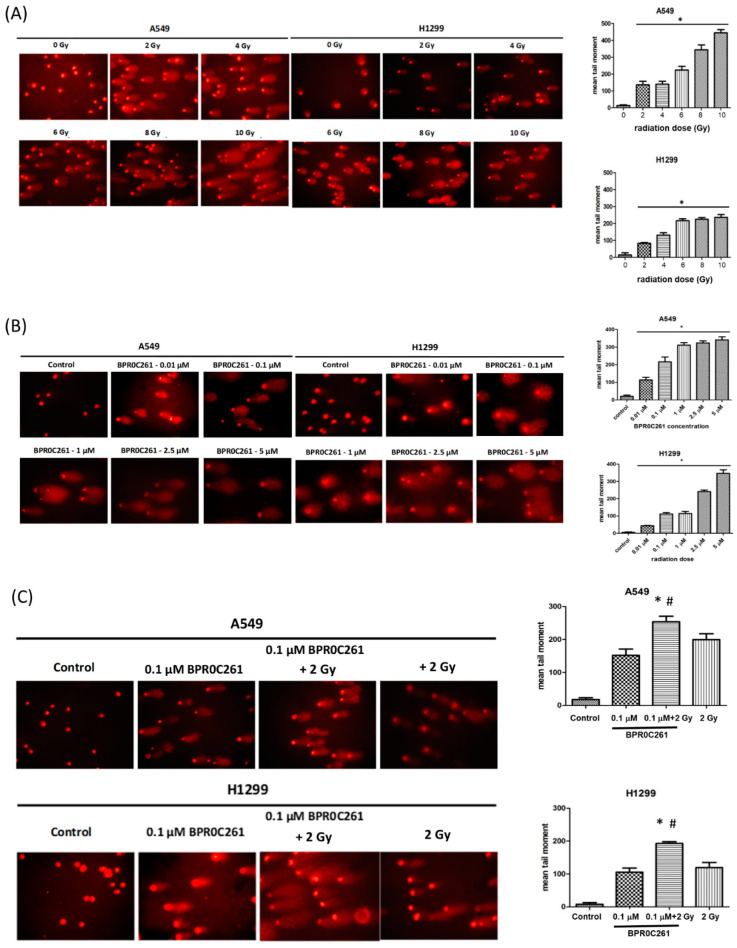
Analysis of DNA damage in NSCLC cells using the comet assay. The images of DNA damage in A549 cells and H1299 cells exposed to (**A**) different doses of X-rays and (**B**) increased concentrations of BPR0C261. The level of DNA damage was quantified by measuring the tail moments. (**C**) Comparison of DNA damage induced by a combination of low concentration of BPR0C261 and 2 Gy X-rays, and individual treatment alone. The microscopic magnification for visualization of comets were 20×. *: *p* < 0.05 compared with BPR0C261 alone. #: *p* < 0.05 compared with radiation alone.

To determine the DNA damage in NSCLC cells treated with BPR0C261 and/or radiation, single-cell gel electrophoresis (comet assay) was exploited for the analysis. The quantification of DNA damage by the comet assay was accomplished by calculating the tail moment. The radiation doses ranging from 2 Gy to 10 Gy showed a dose-dependent increase of DNA damage in A549 cells and H1299 cells (Figure 5A). Surprisingly, the same effect was detected in cells treated with BPR0C261 from 0.01 M to 1 M for 24 h (Figure 5B). We further treated cells with 0.1 M BPR0C261 for 24 h followed by 2 Gy of X-rays exposure. The results showed that this combination induced higher tail moments than the single treatment with BPR0C261 or X-rays on both NSCLC cell lines (Figure 5C). These data indicated that a low concentration of BPR0C261 enhanced the efficacy of a therapeutic radiation dose (2 Gy) for inducing DNA damage.

### 2.6. The Expression of p53 and PTEN in NSCLC Cells Treated with BPR0C261 and Radiation

**Figure 6:**
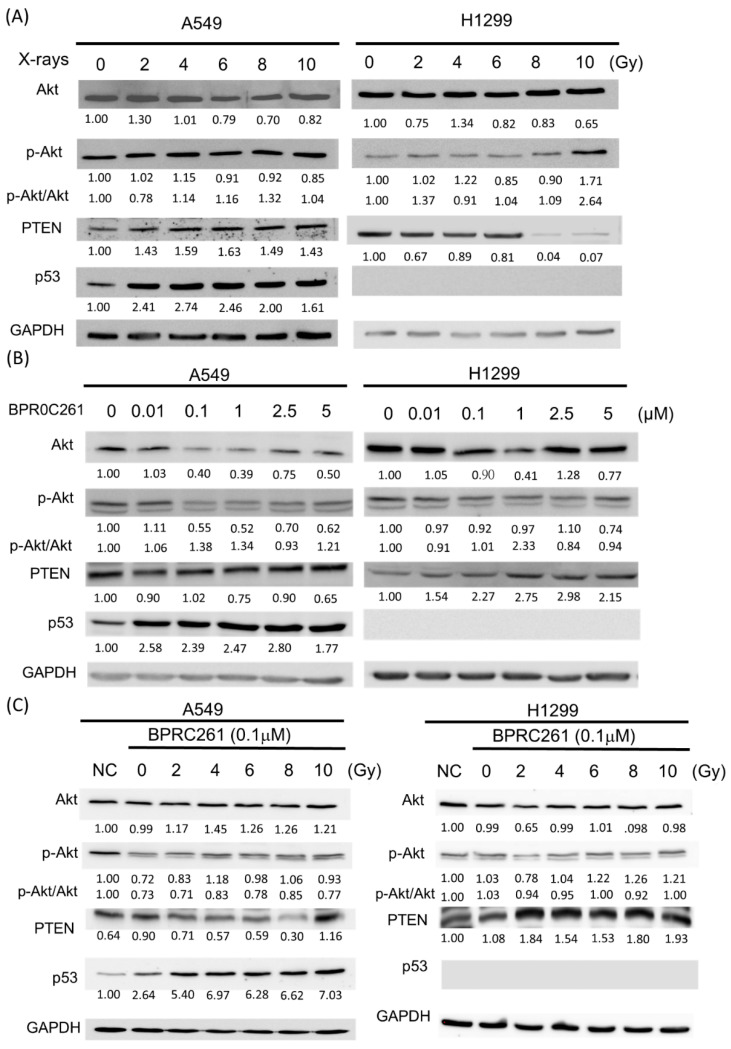
Expression of p53 and PTEN in NSCLC cells treated with BPR0C261 and radiation. (**A**) Western blot analysis of Akt, p-Akt, PTEN, and p53 in A549 cells and H1299 cells exposed to different doses of X-rays. (**B**) Same as (**A**) but treated with different concentrations of BPR0C261. (**C**) A combination of BPR0C261 and X-rays on the expression of proteins described above. NC is the negative control without treatment. The blots of Akt, pAkt, PTEN, and p53 were normalized to that of GAPDH, and then compared to untreated control or NC to determine the ratio of each treatment. The results are from three independent experiments.

As BPR0C261 induced cell cycle arrest and DNA damage in NSCLC cells with or without p53, we next examined the expression of p53 and PTEN mentioned above after cells were treated with BPR0C261 and/or radiation. We first irradiated A549 cells (p53+/+) and H1299 cells (p53-null) from 2 Gy to 10 Gy and showed that p53 was significantly upregulated in A549 cells after radiation exposure, and PTEN was also upregulated (Figure 6A). On the other hand, PTEN expression was downregulated and phosphorylated Akt (p-Akt) was upregulated in p53-null H1299 cells after irradiation over 8 Gy (Figure 6A). We next treated NSCLC cells with BPR0C261 from 0.01 M to 5 M for 24 h. The results showed that BPR0C261 could efficiently upregulate p53 but not PTEN, and it could suppress the expression of Akt protein and p-Akt in A549 cells (Figure 6B). In H1299 cells, PTEN could be upregulated by BPR0C261 but Akt and p-Akt were not affected (Figure 6B). The drug treatment or radiation alone did not dramatically reduce the ratios of p-Akt/Akt. Furthermore, a combination of 0.1 M BPR0C261 and incremental doses of X-rays showed that p53 and PTEN were upregulated in A549 cells and H1299 cells, respectively (Figure 6C). The expression of Akt protein was not significantly affected by the cotreatment of BPR0C261 and radiation. Compared to untreated control, however, the p-Akt levels of A549 cells were more affected than those of H1299 cells after cotreatment of BPR0C261 and radiation.

## 3. Discussion

It has been reported that human NSCLC cells are more resistant to ionizing radiation than small cell lung cancer (SCLC) cells in vitro [27]. Additionally, NSCLC accounts for 80–85% of human lung cancer [28]. Therefore, the development of effective and biocompatible radiosensitizers is still ongoing for the treatment of NSCLC.

In a previous study, we demonstrated that BPR0C123 and BPR0C259 could induce G_2_/M phase arrest in A549 and H1299 cells. However, the significant enhancement of radiosensitivity by these two compounds was only found in NSCLC cells exposed to 10 Gy -rays [9]. Because 2 Gy is routinely used for fractionation radiotherapy in clinics, it is expected to have a radiosensitizer that can raise the effect at low-dose radiation. In this study, we showed that BPR0C261, a well-studied D-24851 derivative [10], could enhance the radiosensitivity at lower radiation doses, especially the p53-null NSCLC cells. Moreover, a low concentration (0.1 M) and high concentration (1 M) of BPR0C261 induced similar radiosensitive effects. This is important because 0.1 M BPR0C261 induced very low cytotoxicity, which is a required criterion for an ideal radiosensitizer [29]. Notably, according to the pattern of survival curves shown in NSCLC cells treated with BPR0C261, it seemed that H1299 cells exhibited stronger responses to X-rays than A549 cells. Because the intrinsic radiosensitivity of A549 cells is higher than that of H1299 cells, this is most likely due to the presence of wild-type p53 that contributes to the radiosensitivity of lung cancer [30].

BPR0C261 is designed to target microtubules and cause antimitotic effect in cancer cells [10]. Thus, it is no doubt that cells treated with this drug will lead to G2/M phase arrest, although the level may be different. The rationale of BPR0C261-induced radiosensitivity is mainly based on the fact that the G_2_/M phase is the most sensitive phase to radiation [31]. BPR0C261 induced G_2_/M arrest in A549 and H1299 cells, but H1299 cells exhibited an earlier accumulation of G_2_/M phase compared to A549 cells. This phenomenon may also be associated with the p53 gene. When cells are exposed to genotoxic agents, p53-dependent G1 phase arrest will be triggered via the transactivation of the p21 gene, a cyclin-dependent kinase inhibitor (CKI) [32,33]. It is speculated that p53 would be activated by BPR0C261 to induce the G1 phase arrest prior to the G_2_/M phase arrest in A549 cells. On the other hand, the p53-null H1299 cells entered the G2/M phase at an earlier time point after cells were treated with BPR0C261. Although p53 is also demonstrated to be important for G2/M phase arrest in normal human fibroblasts [34], BPR0C261-induced G2/M phase arrest does not depend on the p53 status. Therefore, it may further explain the p53-independent induction of radiosensitivity by this compound.

It has been reported that fractional DNA content is a characteristic of apoptosis that can be recognized in a DNA histogram, namely a sub-G1 or hypodiploid subpopulation detected by flow cytometry [35,36]. Because the sub-G1 phase is believed to be composed of apoptotic cells and necrotic cells, it would be interesting to use other apoptotic and necrotic specific biomarkers to evaluate the types of cell death induced by BPR0C261 in the future [37,38,39]. BPR0C261 has been reported to induce apoptosis by observing the increase of DNA fragmentation and positive TUNEL signals in gastric MKN-45 cells [10]. The analysis of the sub-G1 subpopulation in NSCLC cells suggests that current data agree with previous report that BPR0C261 may also induce apoptosis in this cancer type. Although p53 is important for inducing apoptosis [40], BPR0C261 could increase the percentage of the sub-G1 phase in both p53-positive and p53-negative NSCLC cells. Therefore, other cell death mechanisms should also be involved in BPR0C261-mediated cytotoxicity. Notably, the induction of the sub-G1 phase in NSCLC cells was more robust using a high concentration of BPR0C261. However, it could induce similar levels of G2/M phase arrest at low and high concentrations, suggesting that a low concentration of BPR0C261 may be biocompatible for combining with other therapeutic methods. 

The synergistic effect is another important criterion to evaluate an ideal radiosensitizer. For instance, tirapazamine conjugated to gold nanoparticles exhibited a synergistic radiosensitizing effect on human hepatoma HepG2 cells [41]. Codrug-loaded nanoparticles has also been reported to improve the synergistic therapeutic efficacy of chemoradiotherapy [42]. It is interesting to combine this compound with nanoparticles which are usually used for drug carriers and tracking, to passively target tumor in vivo through the enhanced permeability and retention (EPR) effect [43,44]. In the present study, BPR0C261 also performed synergistic radiosensitizing effects on p53-wild-type A549 cells and p53-null H1299 cells based on the method of Valeriote and Carpentier [45,46]. Drug-induced PTEN in p53-null NSCLC cells may be important for this phenomenon as PTEN mutation has recently been reported to decrease radiosensitivity in NSCLC cells [47]. An improvement of the synergistic radiosensitizing effect may be also expected by combining BPR0C261 and nanoparticles for in vivo treatment in the future.

Radiation is known to induce DNA damage, but whether D-24851-related derivatives could influence the integrity of DNA has been little studied. Although a low concentration of BPR0C261 (0.1 M) did not cause significant cytotoxicity, the comet assay showed that this treatment could increase the tail moment in this assay. Actually, the use of 0.01 M BPR0C261 was already sufficient to induce DNA damage. The induction of DNA damage by BPRC261 in both NSCLC cell lines was dose-dependent. Because of this effect, a low concentration of BPR0C261 also increased the clinical dose (2 Gy) radiation-induced DNA damage. The impairment of microtubule dynamics by paclitaxel, a microtubule stabilizer, has been reported to induce DNA damage [48]. To the best of our knowledge, this is the first report showing that BPR0C261, a microtubule inhibitor containing antitumor activity also induces DNA damage. It may explain the role of BPR0C261 as a potent radiosensitizer, and the underlying mechanism would be interesting to be further investigated.

The molecular mechanisms disturbed by BPR0C261 to influence the radiosensitivity and DNA damage have not been fully investigated. We started from two common tumor suppressor genes p53 and PTEN and showed that they could account for the p53-dependent and the p53-independent pathways raised by BPR0C261. However, current data only demonstrated the upregulation of the p53 protein in A549 cells treated with radiation and/or BPR0C261. The activation and function of p53 are mainly determined by post-translational modifications [49]. It would be important to investigate the mechanisms of p53 activation by BPR0C261-mediated radiation responses in the future. As mentioned, we have demonstrated that another two BPR0C derivatives exhibited p53-independent apoptosis [9]. However, the molecules responsible for this phenomenon remain unknown. In this study, PTEN may represent the tumor suppressor gene in p53-independent pathway after cells were treated by BPR0C261, suggesting that PTEN may also play a role in other BPR0C series compounds. Because somatic PTEN mutation is less found in NSCLC [50,51], the induction of PTEN by BPR0C261 may be an important mechanism for this compound to be applied in various lung cancers. We also found that the level of PTEN in p53-null H1299 cells were suppressed by a high dose of radiation (>8 Gy). Because PTEN is a direct downstream target gene transactivated by p53 [22], it would be interesting to further investigate if the maintenance of the PTEN level at a high dose radiation is associated with p53 status. The counterpart of PTEN, named Akt oncoprotein, was also found to be activated by a high dose of radiation. This is consistent with previous reports that radiation can activate the Akt pathway in different cancer types [52,53]. Although the combination of BPR0C261 and radiation induced, respectively, p53 and PTEN in A549 cells and H1299 cells, the expression and activity of Akt was not induced in both cell types compared to untreated control. However, whether the activity of Akt and related signaling are associated with BPR0C261-modulated radiation responses would be interesting to further investigate in the future.

Taken together, BPR0C261 not only decreased cell viability but also induced G2/M phase arrest and DNA damage with a dose-dependent manner in NSCLC cells. BPR0C261 is regarded as a potent radiosensitizer because it showed a synergistic radiosensitizing effect when combining with conventional dose (2 Gy) for fractionation radiotherapy. The underlying mechanism of BPR0C261-induced radiosensitivity is associated with p53 and PTEN molecules in p53-dependent and p53-independent pathways. Current data suggest that BPR0C261 may modulate the effect of radiation on the treatment of lung cancer cells in vitro. To evaluate the radiosensitizing role of BPR0C261 in potent clinical application, an animal study using a xenograft tumor model should be designed soon to confirm the radiosensitizing efficacy of BPR0C261 in vivo.

## 4. Materials and Methods

### 4.1. Cell Culture

Human A549 epithelial adenocarcinoma cells and human lymph node invaded H1299 non-small cell lung carcinoma cells were cultured in Dulbecco’s Modified Eagle Medium (DMEM medium) supplemented with 10% fetal bovine serum (FBS), 1% penicillin–streptomycin solution (P/S), and 1% L-glutamine. Cells were incubated at 37 °C (5% CO_2_ in air) and subcultured by trypsinization every 48 h.

### 4.2. Reagent

BPR0C261 with the chemical formula N-heterocyclic indolyl glyoxylamide N-(3-methyl-5-isothiazolyl)-2-1[(3-methyl-5-isoxazolyl-)methyl]-1H-3-indoyl-2-oxoacetamide was synthesized in the institute of Biotechnology and Pharmaceutical Research, National Health and Research Institute, Taiwan [10]. The stock solution (1 mM) of BPR0C261 was dissolved in dimethyl sulfoxide (DMSO). An appropriate volume of DMEM medium was added to dilute the stock solution for specific experiments.

### 4.3. Radiation Source

Radiation was delivered by the cabinet digital X-ray machine (RS 2000 Biological Research X-ray Irradiator; Rad Source Technologies, Inc., Suwanee, GA, USA) operating at 160 kVp and 25 mA. Cells were suspended in a T-25 flask and exposed to radiation at a dose rate of 38.37 mGy per second.

### 4.4. Cell Viability Assay

For cell viability assays, 4.5 × 10^3^ cells were seeded in each well of a 96-well plate. Different concentrations of BPR0C261 were used to treat cells for 24 h. Subsequently, 0.5 mM 3-(4,5-Dimethylthiazol-2-yl)-2,5-diphenyltetrazolium bromide (MTT) dissolved in serum free DMEM was added (100 μL/well) into each well and incubated at 37 °C for 3 h. DMSO was then used to dissolve the insoluble purple formazan crystals. The absorbance of this solution was quantified at the wavelength of 570 nm by a Tecan’s Sunrise absorbance microplate reader (TECAN Group Ltd., Männedorf, Switzerland). The cell viability was determined by the following formula: (the optical density (OD value) of the experiments group/the OD value of control group) × 100%, and the control group was normalized to 100%. The IC_50_ was calculated using Prism statistical software (Ver.5, GraphPad Software, San Diego, CA, USA). The change of cell viability was also visualized by a bright field microscope (Leica DM IRB, Wetzlar, Germany). The images were captured by a camera (Powershot A260, Canon U.S.A. Inc., New York, NY, USA).

### 4.5. Flow Cytometry Analysis

The cell cycle analysis were executed by following a previous report with slight modification [54]. In brief, A549 cells and H1299 cells (1 × 10^6^ each) were seeded in 10 cm dishes and exposed to radiation or drug. The cells were then rinsed, trypsinized, and collected by centrifugation at 600 rpm for 5 min. To fix the cells, 70% precooled ethanol was added to cell pellets and kept at 4 °C overnight. The cells were then collected and treated with RNase A for 30 min at room temperature. After centrifugation, the cells were resuspended in 20 μg/mL of propidium iodide (PI) and sieved through a 37 m mesh. The DNA histogram was analyzed using a Beckman Coulter Cytomic FC500 flow cytometer and its bundled software (Beckman Coulter, Inc., Bream CA, USA). The sub-G1, G1, S, and G2/M phases were individually gated for quantification.

### 4.6. Colony Formation Assay

The radiosensitivity was determined by calculating the survival fractions of radiation doses. After radiation exposure as mentioned above, the cells were counted and seeded into 6 cm culture dishes and incubated at 37 °C for 14 days. The colonies were stained with 1.25% crystal violet in 100% alcohol. A colony should contain at least 50 cells visualized under a bright field microscope. The formula for the plating efficiency (PE) was: PE = (number of colonies/number of cell seeded) × 100%. Survival fraction (SF) = PE of irradiated cells/PE of unirradiated cells. The survival fractions of drug-combining radiation was measured and assayed according to the method of Valeriote and Carpentier [45,46]. In Table 1 and Table 2, SF_R_ and SF_D_ are the survival fractions of cells treated by radiation and BPR0C261, respectively. SF_R+D_ is the survival fraction of cells treated with radiation and BPR0C261 at different concentrations. The value of SF_R+D_ was compared to the corresponding SF_R_ × SF_D_, and the combination treatment effect was considered synergistic when SF_R+D_ < SF_R_ × SF_D._

### 4.7. Single-Cell Gel Electrophoresis Assay (Comet Assay)

Irradiated cells (1 × 10^4^) were mixed with 75 L 0.75% low melting agarose (LAMDA Biotech Inc., St. Louis, MO, USA). It was loaded onto a slide covered by a spread of 1% normal agarose gel. After solidification, another 1% normal agarose gel was used to spread and cover on the low-melting agarose mixed cells. The prepared slide was immersed in the lysis solution (1% Triton X-100, 2.5 M NaCl, 0.1 M ethylenediaminetetraacetic acid (EDTA) and 10 mM Tris, pH = 10) at 4 °C for 1 h. Cells in the slide were subjected to electrophoresis in the alkaline electrophoresis buffer (300 mM NaOH, 1 mM EDTA and the pH value was adjusted to higher than 12) for 40 min. The condition of electrophoresis was 1 V/cm with a constant 300 mA. The gels were washed three times by the neutralization buffer (0.4 mM Tris, pH 7.5). Cells were then stained using 20 μg/mL of ethidium bromide (EtBr) as a nucleic acid staining reagent and 25 μL of 20 μg/mL EtBr was used and visualized under a fluorescence microscope (Leica DM IRB, Wetslar, Germany). The images were acquired using a digital camera (Powershot A260, Canon U.S.A. Inc.) and quantified by CometScore 2.0 software (Ver. 2.0, The TriTek Corporation, Fairfax, VA, USA). More than 50 cells were counted in each group.

### 4.8. Western Blot Analysis

The Western blot analysis was performed by following a previous report with slight modification [55]. In brief, 30–50 μg of protein lysates were run on the sodium dodecyl sulfate-polyacrylamide gel electrophoresis (SDS-PAGE), and electrotransferred to a nitrocellulose membrane for the detection of specific proteins by primary antibodies. The primary antibodies used in this study included anti-p53 antibody (GTX70214; GeneTex, Inc., Irvine, CA, USA), anti-PTEN antibody (Cat#9552; Cell Signaling Technology^®^, Danvers, MA, USA), anti-Akt antibody (sc-7126), phosphor-specific anti-Akt antibody, (sc-7985, Santa Cruz Biotechnology, Inc., Dallas, TX, USA), and anti-glyceraldehyde-3-phosphate dehydrogenase (GAPDH) antibody (MA5-15738; Thermo Fisher Scientific, Waltham, MA, USA). Enhanced chemiluminescent (ECL) agents (Bio-Rad Laboratories, Hercules, CA, USA) were used for imaging the membrane that was acquired by ImageQuant LAS4000 (GE Healthcare, Buckinghamshire, UK). The blots were quantified using the densitometry involved in ImageJ software (National Institutes of Health, Bethesda, MD, USA).

### 4.9. Statistical Analysis

Each datum is presented as means ± S.D. The statistical analysis was determined using a *t*-test. The statistical significance was defined as *p* < 0.05. Tables were drawn using GraphPad Prism statistical software (Ver.5, GraphPad Software, San Diego, CA, USA).

## Figures and Tables

**Table 1:** Effect of BPR0C261 combining different doses of X-rays on A549 cells.

The SF_D_ of BPR0C261 0.1 μM = 0.368			
Radiation (Gy)	SF_R_	SF_R+D_	SF_R_ × SF_D_
2 Gy	0.4824	0.0822 (synergism)	0.1774
4 Gy	0.1394	0.0264 (synergism)	0.0513
6 Gy	0.0433	0.0056 (synergism)	0.0159
8 Gy	0.0159	0.0020 (synergism)	0.0058
10 Gy	0.0032	0.0003 (synergism)	0.0012
**The SF_D_ of BPR0C261 1 μM = 0.305**			
**Radiation (Gy)**	**SF_R_**	**SF_R+D_**	**SF_R_ × SF_D_**
2 Gy	0.4824	0.1057 (synergism)	0.1471
4 Gy	0.1394	0.0313 (synergism)	0.0425
6 Gy	0.0433	0.0076 (synergism)	0.0132
8 Gy	0.0159	0.0011 (synergism)	0.0048
10 Gy	0.0032	0.0002 (synergism)	0.0010

**Table 2:** Effect of BPR0C261 combining different doses of X-rays on H1299 cells.

The SF_D_ of BPR0C261 0.1 μM = 0.504			
Radiation (Gy)	SF_R_	SF_R+D_	SF_R_ × SF_D_
2 Gy	0.9917	0.3326 (synergism)	0.4998
4 Gy	0.6482	0.2123 (synergism)	0.3267
6 Gy	0.4202	0.0826 (synergism)	0.2118
8 Gy	0.2375	0.0640 (synergism)	0.1197
10 Gy	0.0983	0.0249 (synergism)	0.0495
**The SF_D_ of BPR0C261 1 μM = 0.412**			
**Radiation (Gy)**	**SF_R_**	**SF_R+D_**	**SF_R_ × SF_D_**
2 Gy	0.9917	0.3555 (synergism)	0.4085
4 Gy	0.6482	0.2038 (synergism)	0.2670
6 Gy	0.4202	0.0665 (synergism)	0.1731
8 Gy	0.2375	0.0358 (synergism)	0.0978
10 Gy	0.0983	0.0152 (synergism)	0.0405

## Data Availability

The data presented in this study are available in Supplementary Materials.

## Supplementary Materials

The supporting information can be downloaded at: https://www.mdpi.com/article/10.3390/ijms232214083/s1.
